# Supplementary material for: Hybrid Models and Biological Model Reduction with PyDSTool
Source: PLoS Comput Biol. 2012 Aug 9;8(8):e1002628. doi: 10.1371/journal.pcbi.1002628 (PMC3415397; doi:10.1371/journal.pcbi.1002628)
Supplement: Text S4 — Complete source code for the PyDSTool package (version 0.88.120504). Includes API documentation and help files linking to web pages. This file is identical to the current public release on Sourceforge.net. (ZIP) [file pcbi.1002628.s004.zip › PyDSTool/html/PyDSTool.ModelSpec'-module.html]

xml version="1.0" encoding="ascii"?


PyDSTool.ModelSpec'


| Home | Trees | Indices | Help | PyDSTool |
| --- | --- | --- | --- | --- |

Package PyDSTool ::
Module ModelSpec'

[hide private]

[frames] | no frames]

# Module ModelSpec'

source code

```
Structured model specification classes, and associated utilities.

Robert Clewley, October 2005.


 Technical notes for ModelSpec attributes:
 _componentNameMap: object -> full (hierarchical) objname
 _registry: objname -> regObject:
                      obj, objLocalName, objClassName, objParentName
 _componentTypeMap: classname -> [objlist]
```


|  |  |
| --- | --- |
| Classes | [hide private] |

Component  
Non-leaf node sub-class of ModelSpec abstract class.

LeafComponent  
Leaf node sub-class of ModelSpec abstract class.

ModelSpec  
Model specification abstract class.

nameResolverClass  
This class keeps a tab of how many times a local name has been used
for a given specfication type ('var', 'par', 'input' or 'auxfn'),
and renames it with an appropriate globalized name (hierarchical
according to declared parent object, with possible numbered suffix
for multiple name declarations).

regObject  
Registry object container.

typeCounter


|  |  |
| --- | --- |
| Functions | [hide private] |

|  |  |
| --- | --- |
| accrueCompTypes(c)  Collect all super-class component types (string names) and accumulate recursively. | source code |

|  |  |
| --- | --- |
| allCompTypes(class\_list)  Filter full list of all super-classes' compatible sub-component types to remove any classes that are not over-ridden by sub-classes. | source code |

|  |  |
| --- | --- |
| matchSubName(nameList, subName, position, level=0, invert=False)  Return a list of names from nameList matching subName at the given position (at the hierarchical name level given, if the name is hierarchical). | source code |

|  |  |
| --- | --- |
| processMacro(c, mspec, forSubs=False)  Process 'for' macro occurrences in a Quantity specification. | source code |

|  |  |
| --- | --- |
| resolveMacroTargets(toks, mspec, targParent) | source code |

|  |  |
| --- | --- |
| searchModelSpec(mspec, name, component\_type\_order=None)  Find Quantity or sub-component objects containing named <name> of type given by the hierarchical name <comptype1.comptype2. | source code |


|  |  |
| --- | --- |
| Variables | [hide private] |

API = `API_class()`

Abs = `Abs (ModelSpec wrapper)`

Acos = `Acos (ModelSpec wrapper)`

Asin = `Asin (ModelSpec wrapper)`

Atan = `Atan (ModelSpec wrapper)`

Atan2 = `Atan2 (ModelSpec wrapper)`

Betavariate = `Betavariate (ModelSpec wrapper)`

Ceil = `Ceil (ModelSpec wrapper)`

Choice = `Choice (ModelSpec wrapper)`

Continuous = `Continuous Domain`

Cos = `Cos (ModelSpec wrapper)`

Cosh = `Cosh (ModelSpec wrapper)`

Degrees = `Degrees (ModelSpec wrapper)`

Discrete = `Discrete Domain`

E = `QuantSpec e (ExpFuncSpec)`

Exp = `Exp (ModelSpec wrapper)`

Expovariate = `Expovariate (ModelSpec wrapper)`

Fabs = `Fabs (ModelSpec wrapper)`

Floor = `Floor (ModelSpec wrapper)`

Fmod = `Fmod (ModelSpec wrapper)`

Frexp = `Frexp (ModelSpec wrapper)`

Gammavariate = `Gammavariate (ModelSpec wrapper)`

Gauss = `Gauss (ModelSpec wrapper)`

Getrandbits = `Getrandbits (ModelSpec wrapper)`

Getstate = `Getstate (ModelSpec wrapper)`

Hypot = `Hypot (ModelSpec wrapper)`

Inf = `inf`

Jumpahead = `Jumpahead (ModelSpec wrapper)`

LargestInt32 = `2147483647`

Ldexp = `Ldexp (ModelSpec wrapper)`

Log = `Log (ModelSpec wrapper)`

Log10 = `Log10 (ModelSpec wrapper)`

Lognormvariate = `Lognormvariate (ModelSpec wrapper)`

Max = `Max (ModelSpec wrapper)`

Min = `Min (ModelSpec wrapper)`

Modf = `Modf (ModelSpec wrapper)`

NAMESEP = `'.'`

NaN = `nan`

Normalvariate = `Normalvariate (ModelSpec wrapper)`

ONES = `['1', '1.0', '1.', '(1)', '(1.0)', '(1.)']`

Paretovariate = `Paretovariate (ModelSpec wrapper)`

Pi = `QuantSpec pi (ExpFuncSpec)`

Pow = `Pow (ModelSpec wrapper)`

Radians = `Radians (ModelSpec wrapper)`

Randint = `Randint (ModelSpec wrapper)`

Random = `Random (ModelSpec wrapper)`

Randrange = `Randrange (ModelSpec wrapper)`

Sample = `Sample (ModelSpec wrapper)`

Seed = `Seed (ModelSpec wrapper)`

Setstate = `Setstate (ModelSpec wrapper)`

Shuffle = `Shuffle (ModelSpec wrapper)`

Sin = `Sin (ModelSpec wrapper)`

Sinh = `Sinh (ModelSpec wrapper)`

Sqrt = `Sqrt (ModelSpec wrapper)`

Sum = `Sum (ModelSpec wrapper)`

Systemrandom = `Systemrandom (ModelSpec wrapper)`

Tan = `Tan (ModelSpec wrapper)`

Tanh = `Tanh (ModelSpec wrapper)`

Uniform = `Uniform (ModelSpec wrapper)`

Vonmisesvariate = `Vonmisesvariate (ModelSpec wrapper)`

Weibullvariate = `Weibullvariate (ModelSpec wrapper)`

Wichmannhill = `Wichmannhill (ModelSpec wrapper)`

ZEROS = `['0', '0.0', '0.', '(0)', '(0.0)', '(0.)']`

\_1DimplicitSolveMethods = `['newton', 'bisect', 'steffe']`

\_all\_complex = `(<type 'complex'>, <type 'numpy.complexfloating...`

\_all\_float = `(<type 'float'>, <type 'numpy.floating'>, <type '...`

\_all\_int = `(<type 'int'>, <type 'numpy.integer'>, <type 'numpy...`

\_all\_numpy\_complex = `(<type 'numpy.complex128'>, <type 'numpy....`

\_all\_numpy\_float = `(<type 'numpy.float64'>, <type 'numpy.float...`

\_all\_numpy\_int = `(<type 'numpy.int32'>, <type 'numpy.int32'>, ...`

\_classes = `['ModelSpec', 'Component', 'LeafComponent', 'nameRe...`

\_complex\_types = `(<type 'complex'>, <type 'numpy.complexfloati...`

\_float\_types = `(<type 'float'>, <type 'numpy.floating'>)`

\_functions = `['searchModelSpec', 'matchSubName', 'processMacro...`

\_implicitSolveMethods = `['newton', 'bisect', 'steffe', 'fsolve']`

\_indentstr = `' '`

\_int\_types = `(<type 'int'>, <type 'numpy.integer'>)`

\_num\_equivtype = `{<type 'float'>: <type 'numpy.float64'>, <typ...`

\_num\_maxmin = `{<type 'numpy.int32'>: [-2147483648, 2147483647]...`

\_num\_name2equivtypes = `{'float': (<type 'float'>, <type 'numpy...`

\_num\_name2type = `{'float': <type 'numpy.float64'>, 'int': <typ...`

\_num\_type2name = `{<type 'float'>: 'float', <type 'int'>: 'int'...`

\_num\_types = `(<type 'float'>, <type 'int'>, <type 'numpy.float...`

\_objects = `['nameResolver']`

\_pytypefromtype = `{<type 'numpy.int32'>: <type 'int'>, <type '...`

\_real\_types = `(<type 'int'>, <type 'numpy.integer'>, <type 'fl...`

\_seq\_types = `(<type 'list'>, <type 'tuple'>, <type 'numpy.ndar...`

allmathnames = `['acos', 'asin', 'atan', 'atan2', 'ceil', 'cos'...`

allmathnames\_symbolic = `['Acos', 'Asin', 'Atan', 'Atan2', 'Cei...`

builtinFnSigInfo = `{'for': 4, 'getbound': 2, 'getindex': 1, 'g...`

builtin\_auxnames = `['globalindepvar', 'initcond', 'heav', 'if'...`

e = `2.71828182846`

inverseMathNameMap = `{'Random': 'Random', 'SystemRandom': 'Sys...`

isfinite = `<ufunc 'isfinite'>`

mathNameMap = `{'Abs': 'abs', 'Acos': 'acos', 'Asin': 'asin', '...`

mod = `<ufunc 'remainder'>`

nameResolver = `ModelSpec internal helper class: nameResolver o...`

name\_chars\_RE = `re.compile(r'\w')`

null\_predicate = `null_predicate_class(None)`

num\_chars = `['0', '1', '2', '3', '4', '5', '6', '7', '8', '9']`

pi = `3.14159265359`

protected\_allnames = `['acos', 'asin', 'atan', 'atan2', 'ceil',...`

protected\_auxnamesDB = `ModelSpec internal helper class: auxfnD...`

protected\_macronames = `['for', 'if', 'max', 'min', 'sum']`

protected\_mathnames = `['acos', 'asin', 'atan', 'atan2', 'ceil'...`

protected\_randomnames = `['BPF', 'LOG4', 'NV_MAGICCONST', 'RECI...`

protected\_scipynames = `['sign', 'mod']`

protected\_specialfns = `['special_airy', 'special_airye', 'spec...`

specTypes = `('RHSfuncSpec', 'ImpFuncSpec', 'ExpFuncSpec')`

syms = `{0: 'ENDMARKER', 1: 'NAME', 2: 'NUMBER', 3: 'STRING', 4...`

targetLangs = `['c', 'python', 'matlab']`


|  |  |
| --- | --- |
| Function Details | [hide private] |

|  |  |
| --- | --- |
| allCompTypes(class\_list) | source code |

Filter full list of all super-classes' compatible sub-component types
to remove any classes that are not over-ridden by sub-classes.

e.g. If Par was allowed for Component type, but a sub-class of
Component specifies only a sub-class of Par may be used, then Par is
removed from the original list of valid component types.

|  |  |
| --- | --- |
| matchSubName(nameList, subName, position, level=0, invert=False) | source code |

Return a list of names from nameList matching subName at the given
position (at the hierarchical name level given, if the name is
hierarchical).

e.g. For a hierarchical variable name such as 'sRM.s\_cell1\_cell2',
level=0 will cause a search for a match with subName in the top-level
name, i.e. 'sRM', at the given position. Whereas, level=1 will search for
a match in 's\_cell1\_cell2' at the given position.

invert=True will return names that did not match.

|  |  |
| --- | --- |
| processMacro(c, mspec, forSubs=False) | source code |

Process 'for' macro occurrences in a Quantity specification.

forSubs = True causes any target names to be resolved to their global
names and returned to caller so that they can be substituted textually
into the Quantity's specification.

|  |  |
| --- | --- |
| searchModelSpec(mspec, name, component\_type\_order=None) | source code |

Find Quantity or sub-component objects containing named <name>
of type given by the hierarchical name <comptype1.comptype2. ...
.name>, where component\_type\_order = [<comptype1>,
<comptype2>, ... ], and <comptypeN> may be a component type
or a specific component name.

  


|  |  |
| --- | --- |
| Variables Details | [hide private] |

### \_all\_complex

Value:
:   ```
    (<type 'complex'>,
     <type 'numpy.complexfloating'>,
     <type 'numpy.complex128'>,
     <type 'numpy.complex64'>,
     <type 'numpy.complex128'>)
    ```

### \_all\_float

Value:
:   ```
    (<type 'float'>,
     <type 'numpy.floating'>,
     <type 'numpy.float64'>,
     <type 'numpy.float32'>,
     <type 'numpy.float64'>)
    ```

### \_all\_int

Value:
:   ```
    (<type 'int'>,
     <type 'numpy.integer'>,
     <type 'numpy.int32'>,
     <type 'numpy.int32'>,
     <type 'numpy.int8'>,
     <type 'numpy.int16'>,
     <type 'numpy.int32'>,
     <type 'numpy.int64'>)
    ```

### \_all\_numpy\_complex

Value:
:   ```
    (<type 'numpy.complex128'>,
     <type 'numpy.complex64'>,
     <type 'numpy.complex128'>)
    ```

### \_all\_numpy\_float

Value:
:   ```
    (<type 'numpy.float64'>,
     <type 'numpy.float32'>,
     <type 'numpy.float64'>)
    ```

### \_all\_numpy\_int

Value:
:   ```
    (<type 'numpy.int32'>,
     <type 'numpy.int32'>,
     <type 'numpy.int8'>,
     <type 'numpy.int16'>,
     <type 'numpy.int32'>,
     <type 'numpy.int64'>)
    ```

### \_classes

Value:
:   ```
    ['ModelSpec', 'Component', 'LeafComponent', 'nameResolverClass']
    ```

### \_complex\_types

Value:
:   ```
    (<type 'complex'>, <type 'numpy.complexfloating'>)
    ```

### \_functions

Value:
:   ```
    ['searchModelSpec',
     'matchSubName',
     'processMacro',
     'resolveMacroTargets']
    ```

### \_num\_equivtype

Value:
:   ```
    {<type 'float'>: <type 'numpy.float64'>,
     <type 'int'>: <type 'numpy.int32'>,
     <type 'numpy.integer'>: <type 'numpy.int32'>,
     <type 'numpy.floating'>: <type 'numpy.float64'>,
     <type 'numpy.int8'>: <type 'numpy.int32'>,
     <type 'numpy.int16'>: <type 'numpy.int32'>,
     <type 'numpy.int32'>: <type 'numpy.int32'>,
     <type 'numpy.int32'>: <type 'numpy.int32'>,
    ...
    ```

### \_num\_maxmin

Value:
:   ```
    {<type 'numpy.int32'>: [-2147483648, 2147483647],
     <type 'numpy.float64'>: [-inf, inf]}
    ```

### \_num\_name2equivtypes

Value:
:   ```
    {'float': (<type 'float'>,
               <type 'numpy.floating'>,
               <type 'numpy.float64'>,
               <type 'numpy.float32'>,
               <type 'numpy.float64'>),
     'int': (<type 'int'>,
             <type 'numpy.integer'>,
             <type 'numpy.int32'>,
    ...
    ```

### \_num\_name2type

Value:
:   ```
    {'float': <type 'numpy.float64'>, 'int': <type 'numpy.int32'>}
    ```

### \_num\_type2name

Value:
:   ```
    {<type 'float'>: 'float',
     <type 'int'>: 'int',
     <type 'numpy.integer'>: 'int',
     <type 'numpy.floating'>: 'float',
     <type 'numpy.int8'>: 'int',
     <type 'numpy.int16'>: 'int',
     <type 'numpy.int32'>: 'int',
     <type 'numpy.int32'>: 'int',
    ...
    ```

### \_num\_types

Value:
:   ```
    (<type 'float'>,
     <type 'int'>,
     <type 'numpy.floating'>,
     <type 'numpy.integer'>)
    ```

### \_pytypefromtype

Value:
:   ```
    {<type 'numpy.int32'>: <type 'int'>,
     <type 'numpy.float64'>: <type 'float'>}
    ```

### \_real\_types

Value:
:   ```
    (<type 'int'>,
     <type 'numpy.integer'>,
     <type 'float'>,
     <type 'numpy.floating'>)
    ```

### \_seq\_types

Value:
:   ```
    (<type 'list'>, <type 'tuple'>, <type 'numpy.ndarray'>)
    ```

### allmathnames

Value:
:   ```
    ['acos',
     'asin',
     'atan',
     'atan2',
     'ceil',
     'cos',
     'cosh',
     'degrees',
    ...
    ```

### allmathnames\_symbolic

Value:
:   ```
    ['Acos',
     'Asin',
     'Atan',
     'Atan2',
     'Ceil',
     'Cos',
     'Cosh',
     'Degrees',
    ...
    ```

### builtinFnSigInfo

Value:
:   ```
    {'for': 4,
     'getbound': 2,
     'getindex': 1,
     'globalindepvar': 1,
     'heav': 1,
     'if': 3,
     'initcond': 1,
     'max': 1,
    ...
    ```

### builtin\_auxnames

Value:
:   ```
    ['globalindepvar', 'initcond', 'heav', 'if', 'getindex', 'getbound']
    ```

### inverseMathNameMap

Value:
:   ```
    {'Random': 'Random',
     'SystemRandom': 'Systemrandom',
     'WichmannHill': 'Wichmannhill',
     'abs': 'Abs',
     'acos': 'Acos',
     'asin': 'Asin',
     'atan': 'Atan',
     'atan2': 'Atan2',
    ...
    ```

### mathNameMap

Value:
:   ```
    {'Abs': 'abs',
     'Acos': 'acos',
     'Asin': 'asin',
     'Atan': 'atan',
     'Atan2': 'atan2',
     'Betavariate': 'betavariate',
     'Ceil': 'ceil',
     'Choice': 'choice',
    ...
    ```

### nameResolver

Value:
:   ```
    ModelSpec internal helper class: nameResolver object
    ```

### protected\_allnames

Value:
:   ```
    ['acos',
     'asin',
     'atan',
     'atan2',
     'ceil',
     'cos',
     'cosh',
     'degrees',
    ...
    ```

### protected\_auxnamesDB

Value:
:   ```
    ModelSpec internal helper class: auxfnDBclass object
    ```

### protected\_mathnames

Value:
:   ```
    ['acos',
     'asin',
     'atan',
     'atan2',
     'ceil',
     'cos',
     'cosh',
     'degrees',
    ...
    ```

### protected\_randomnames

Value:
:   ```
    ['BPF',
     'LOG4',
     'NV_MAGICCONST',
     'RECIP_BPF',
     'Random',
     'SG_MAGICCONST',
     'SystemRandom',
     'TWOPI',
    ...
    ```

### protected\_specialfns

Value:
:   ```
    ['special_airy',
     'special_airye',
     'special_ai_zeros',
     'special_bi_zeros',
     'special_ellipj',
     'special_ellipk',
     'special_ellipkinc',
     'special_ellipe',
    ...
    ```

### syms

Value:
:   ```
    {0: 'ENDMARKER',
     1: 'NAME',
     2: 'NUMBER',
     3: 'STRING',
     4: 'NEWLINE',
     5: 'INDENT',
     6: 'DEDENT',
     7: 'LPAR',
    ...
    ```

  


| Home | Trees | Indices | Help | PyDSTool |
| --- | --- | --- | --- | --- |

|  |  |
| --- | --- |
| Generated by Epydoc 3.0.1 on Fri May 4 15:24:03 2012 | http://epydoc.sourceforge.net |
